# Comparison of Clinical Outcome Between Twice-Weekly and Thrice-Weekly Hemodialysis in Patients With Residual Kidney Function

**DOI:** 10.1097/MD.0000000000002767

**Published:** 2016-02-18

**Authors:** Hyeon Seok Hwang, Yoo Ah Hong, Hye Eun Yoon, Yoon Kyung Chang, Suk Young Kim, Young Ok Kim, Dong Chan Jin, Su-Hyun Kim, Yong-Lim Kim, Yon-Su Kim, Shin-Wook Kang, Nam-Ho Kim, Chul Woo Yang

**Affiliations:** From the Department of Internal Medicine, College of Medicine, The Catholic University of Korea (HSH, YAH, HEY, YKC, SYK, YOK, DCJ, YKK, CWY); Department of Internal Medicine, College of Medicine, Chung-Ang University, Seoul (S-HK); Department of Internal Medicine, School of Medicine, Kyungpook National University, Daegu (YLK); Department of Internal Medicine, College of Medicine, Seoul National University (YSK); Department of Internal Medicine, College of Medicine, Yonsei University, Seoul (SWK); and Department of Internal Medicine, Chonnam National University Medical School, Gwangju, Korea (NHK).

## Abstract

Residual kidney function (RKF) contributes to improved survival in hemodialysis (HD) patients. However, it is not clear whether RKF allows a comparable survival rate in patients undergoing twice-weekly HD compared with thrice-weekly HD.

We enrolled 685 patients from a prospective multicenter observational cohort. RKF and HD adequacy was monitored regularly over 3-year follow-up. Patients with RKF were divided into groups undergoing twice-weekly HD (n = 113) or thrice-weekly HD (n = 137). Patients without RKF undergoing thrice-weekly HD (n = 435) were included as controls. Fluid balance and dialysis-associated characteristics were followed and clinical outcomes evaluated using all-cause mortality and cardiovascular events (CVE).

In patients with RKF, baseline and follow-up RKF were significantly higher in patients undergoing twice-weekly HD than in those undergoing thrice-weekly HD. Total Kt/V urea (dialysis plus residual renal) in patients with RKF undergoing twice-weekly HD was greater than or equal to those in patients with or without RKF undergoing thrice-weekly HD. Compared with patients with RKF undergoing thrice-weekly HD, patients with RKF undergoing twice-weekly HD had no fluid excess, but their normalized protein catabolic rate became lower since 24-month follow up. In multivariable analyses, patients with RKF undergoing twice-weekly HD had a noninferior risk of mortality (hazard ratio [HR], 0.83; 95% confidence interval [95% CI], 0.34–2.01, *P* = 0.68) and of CVE (HR, 0.60; 95% CI, 0.28–1.29, *P* = 0.19) compared with patients without RKF undergoing thrice-weekly HD. However, this group showed an independent association with a greater risk of mortality compared with patients with RKF undergoing thrice-weekly HD (HR, 4.20; 95% CI, 1.02–17.32, *P* = 0.04).

In conclusion, patients with RKF undergoing twice-weekly HD had an increased risk of mortality compared with those undergoing thrice-weekly HD. Decisions about twice-weekly HD should consider not only RKF, but also other risk factors such as normalized protein catabolic rate.

## INTRODUCTION

Residual kidney function (RKF) in hemodialysis (HD) patients plays a crucial role in the achievement of HD adequacy.^1^ The solute kinetic effect of RKF is so strong that it can remove large amounts of uremic toxins.^2^ In addition, RKF has been associated with several beneficial effects, including more balanced salt and water removal, more effective phosphorus excretion, and better endogenous vitamin D and erythropoietin production.^3^ Therefore, RKF contributes considerably to the improvement of survival and to cardiovascular protective effects.^4^

In many parts of the world, a dialysis frequency of 3 times per week is the current standard practice to achieve an adequate dialysis dose, and the Kidney Disease Outcomes Quality Initiative (KDOQI) guideline recommends thrice-weekly treatment in patients without RKF. However, a reduction in the frequency of dialysis can be considered in patients with substantial RKF.^5^ Indeed, there are some reports that twice-weekly dialysis does not increase mortality risk in comparison with thrice-weekly therapy, and it is suggested that a twice-weekly regimen is a valid option in cases of HD patients with RKF.^6,7^ However, in these reports, RKF was not evaluated or was estimated only by serum creatinine level. Urine collection, its follow-up data, and dialysis dose including RKF was not investigated.

Therefore, we attempted to evaluate, using regular 24-h urine collection, the clinical outcome of twice-weekly HD for patients with RKF. We examined the effect of RKF on urea removal and fluid balance, and investigated whether substantial RKF allows a comparable outcome for patient survival and cardiovascular events (CVE) in patients undergoing twice-weekly HD compared with those undergoing thrice-weekly HD.

## METHODS

### Study Population

All patients enrolled to the study were on the Clinical Research Center registry for end-stage renal disease (CRC for ESRD). The CRC for ESRD is a nationwide, multicenter, Internet-based, prospective cohort of dialysis patients in Korea (clinicaltrial.gov NCT00931970). Enrolment commenced in April 2009, and included adult (>18 years of age) ESRD patients on dialysis from 31 hospitals. All patients were informed about the study and participated voluntarily with written consent. The study was approved by the institutional review board at each center.

We screened the regular HD patients from the CRC for ESRD cohort who had been receiving HD for >3 months. Patients who were missing urine collection, single-pool Kt/V (spKt/V), or HD frequency at baseline were excluded. A total of 685 regular HD patients were enrolled in this study. The presence of RKF was defined as the production of more than 100 mL/day of urine.^8^ Patients were categorized into twice-weekly HD with RKF, thrice-weekly HD with RKF, and thrice-weekly HD without RKF. Patients without RKF undergoing twice-weekly HD or patients undergoing once-weekly HD were not included. All patients received the in-center HD treatment, and 4-h dialysis per session was prescribed. The dialysis schedule of thrice-weekly HD was Mondays, Wednesdays and Fridays or Tuesdays, Thursdays and Saturdays. The patients undergoing twice-weekly HD received the dialysis on Mondays and Thursdays or Tuesdays and Fridays.

### Data Collection and Definitions

Baseline information at enrollment included age, sex, height, weight, primary renal disease, comorbidities, Davies comorbidity score, laboratory data, and dialysis information. The Davies comorbidity score classifies patients with a score of zero as low risk, patients with a score of 1 to 2 as moderate risk, and patients with a score of 3 as high risk.^9,10^ Scoring comorbidity includes the conditions in the following 7 domains: ischemic heart disease, diabetes mellitus, left ventricular dysfunction, peripheral and cerebral vascular disease, noncutaneous active malignancies, systemic collagen vascular disease and a group of other chronic conditions such as chronic infections, chronic obstructive pulmonary disease, heart valve dysfunctions and liver cirrhosis, and other conditions with poor survival prognosis compared with the general population.

Clinical outcomes, laboratory data, and dialysis information were analyzed. The estimated glomerular filtration rate (eGFR, mL/min per 1.73 m^2^) was calculated using the abbreviated Modification of Diet in Renal Disease formula.^11^ The 24-h urine collection and delivered sp*Kt*/*V* (*K*, dialyzer clearance; *t*, time; *V*, urea distribution volume) were assessed every year. Dates and causes of death during the follow-up period were immediately reported and confirmed by data from Statistics Korea. To assess the impact of RKF and HD frequency on clinical outcomes, follow-up of the patients was censored for all patients if the frequency of HD per week changed or for patients with RKF undergoing twice-weekly or thrice-weekly HD if urine volume decreased to less than 100 mL/day.

Blood urea nitrogen was measured in the urine that was collected during the interdialytic interval. For the calculation of the renal *Kt*/*V*, the mean of the urea concentration in a blood sample collected immediately after the HD session and the urea concentration in a sample collected before the HD session was used. The *V* was determined according to the Watson formula.^12^ The mean serum urea was considered mean value of predialysis and postdialysis serum urea,^1,13^ and following formula was used to calculate RKF and renal *Kt*/*V*.^14^ 
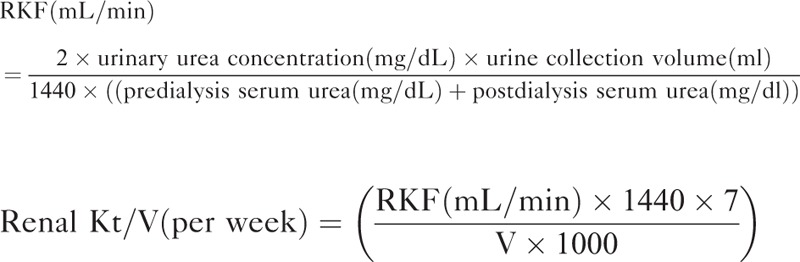


Measurement of RKF was corrected for 1.73-m^2^ body surface area. The sp*Kt*/*V* was determined using 2-point urea modeling based on the intradialytic reduction in blood urea and intradialytic weight loss. To compare the dialysis adequacy for different HD frequencies, standard *Kt*/*V* (std*Kt*/*V*) was calculated from the following formula.^5^ 
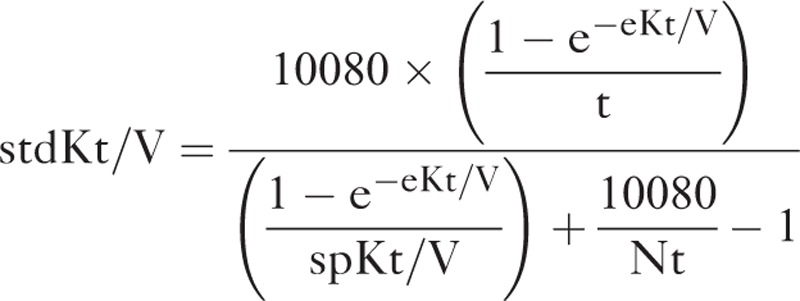


The equilibrated *Kt*/*V* (e*Kt*/*V*) was estimated from sp*Kt*/*V* using the Tattersall method, and total *Kt*/*V* per week was calculated by adding std*Kt*/*V* to the intermittent equivalent of the continuous residual renal urea clearance (renal *Kt*/*V*).^5,15^

Total fluid removal per week was defined as the sum of ultrafiltration per week and weekly urine volume. The following equation was used. 
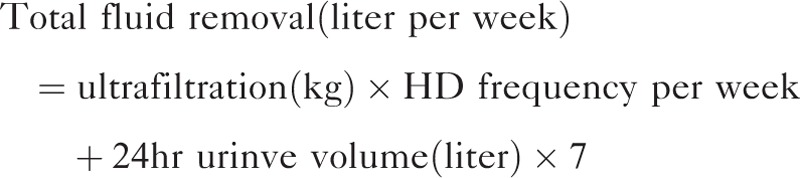


We subtracted the dry weight from the body weight after the dialysis session, and this was defined as the net fluid balance. The protein catabolic rate was calculated on the basis of total urea removed by dialysis, residual renal urea clearance, and protein loss in urine, and was normalized to actual body weight (normalized protein catabolic rate, nPCR).^1^ 
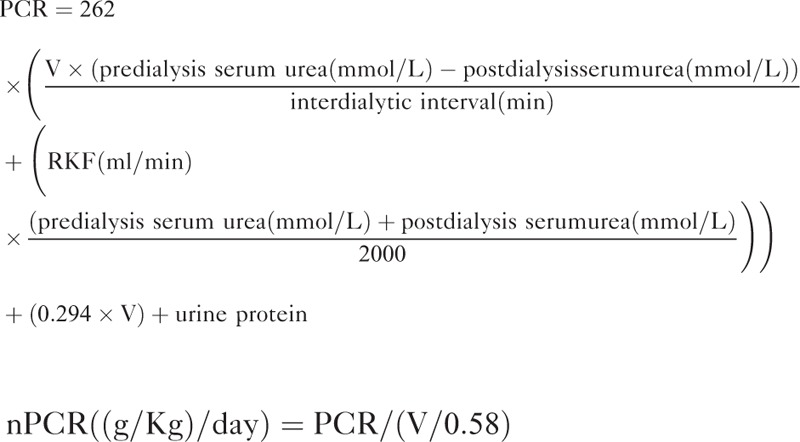


### Outcome Measures

The primary endpoint was all-cause mortality during the 3-year follow-up period. The secondary outcomes included the first hospitalization for CVE. Hospitalization was defined as admission for at least 24 h, including for ischemic heart disease, congestive heart failure, arrhythmia, peripheral vascular disease, and cerebrovascular disease.

### Statistical Analysis

Variables are presented as the mean ± standard deviation (SD) or as frequencies (percentage). Continuous variables were analyzed using Student *t* test or ANOVA followed by Tukey *post-hoc* test. Kolmogorov–Smirnov was used for testing normality. Nonparametric variables were compared using Mann–Whitney *U* or Kruskal–Wallis tests as appropriate. The *χ*^2^ test or Fisher exact test was used for categorical variables.

To test for an independent association of HD frequency and the occurrence of an endpoint, a Cox proportional hazard model was used. Independent variables were selected according to their weight on univariate testing. Baseline clinical, biochemical and dialysis adequacy with a *P* value <0.10, and the established clinical predictors were also included. The confounders entered into the analysis for mortality were age (1-year increments), risk group according to the Davies comorbidity score, body mass index (1 kg/m^2^ increments), duration of dialysis (1-month increments), smoking, predialysis systolic blood pressure (SBP, 1 mm Hg increments), use of erythropoietin-stimulating agent (ESA), hemoglobin (1 g/dL increments), serum levels of albumin (1 g/dL increments), calcium (per 1 mg/dL increments), phosphorus (per 1 mg/dL increments) and intact parathyroid hormone (per 1 pg/mL increments), total fluid removal per week (1 L increments), total *Kt*/*V* per week (0.01 increments), nPCR (0.1 g/kg/day increments), and type of vascular access and dialyzer membrane type. Age, risk group according to Davies comorbidity score, duration of dialysis, predialysis SBP, hemoglobin, use of ESA, serum levels of calcium, phosphorus, and intact parathyroid hormone were entered to analyze the hospitalizations for CVEs. The cumulative event rates for the prespecified endpoint were estimated by the Kaplan–Meier method and compared with log-rank tests. A *P* value of <0.05 was considered to indicate a significant difference. The statistical analysis was performed using SPSS software (version 20.0; IBM, Armonk, NY).

We performed power calculations to estimate the sample size using standard formulas. The number of patients required to show a different patient survival was calculated in patients with RKF undergoing twice-weekly HD or thrice-weekly HD (with α error = 0.05; β error = 0.20; hazard ratio = 1.5). The minimal required number of patients was 98 in each group.

## RESULTS

### Baseline Characteristics in Patients Receiving Twice-Weekly and Thrice-Weekly HD

Of the total HD patients, 113 (16.5%) patients with RKF received twice-weekly HD treatment, and 137 (20.0%) patients with RKF underwent thrice-weekly HD treatment. Table 1 shows the demographics and laboratory values of the study population. Compared with patients without RKF receiving thrice-weekly HD, patients with RKF receiving twice-weekly HD were younger and had a shorter duration of dialysis and lower SBP and diastolic blood pressure. For patients with RKF, the serum calcium level was significantly lower in those receiving twice-weekly HD than in those undergoing thrice-weekly HD. The low flux dialyzer and catheter as the vascular access was used more frequently in patients with RKF undergoing twice-weekly HD.

**TABLE 1:**
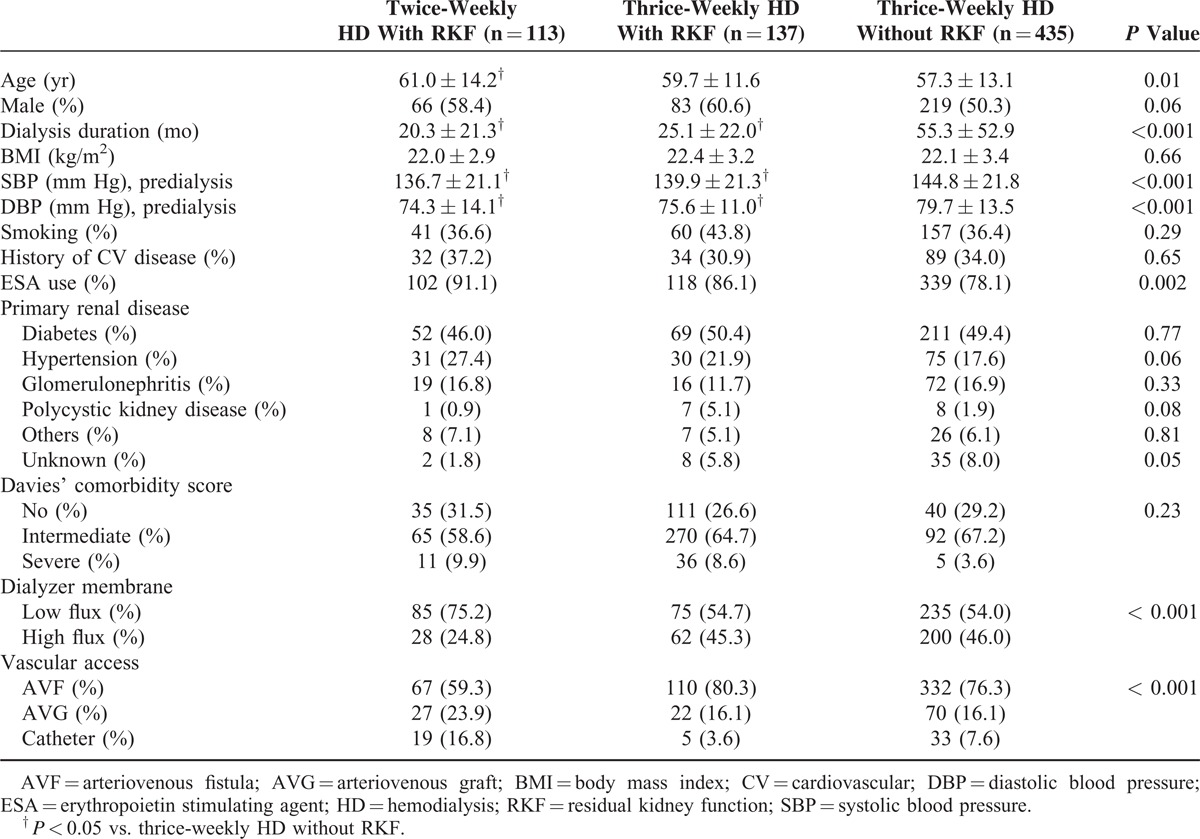
Baseline Demographic and Clinical Characteristics in HD Patients

### RKF and Dialysis Adequacy in Patients Receiving Twice-Weekly and Thrice-Weekly HD

The baseline eGFR of patients with RKF was significantly higher than those without RKF. RKF and renal *Kt*/*V* at all follow-up points was significantly greater in patients with RKF receiving twice-weekly HD than in patients with or without RKF receiving thrice-weekly HD (Table 2). The std*Kt*/*V* values in patients with RKF undergoing twice-weekly HD were significantly lower than those in patients with or without RKF undergoing thrice-weekly HD. Total *Kt*/*V* (renal *Kt*/*V* plus std*Kt*/*V*) in patients with RKF undergoing twice-weekly HD was greater than or equal to those in patients with or without RKF undergoing thrice-weekly HD at all follow-up points. Compared with patients without RKF receiving thrice-weekly HD, total *Kt*/*V* was greater at baseline in patients with RKF undergoing thrice-weekly HD, and remained higher until the 12-month follow-up.

**TABLE 2:**
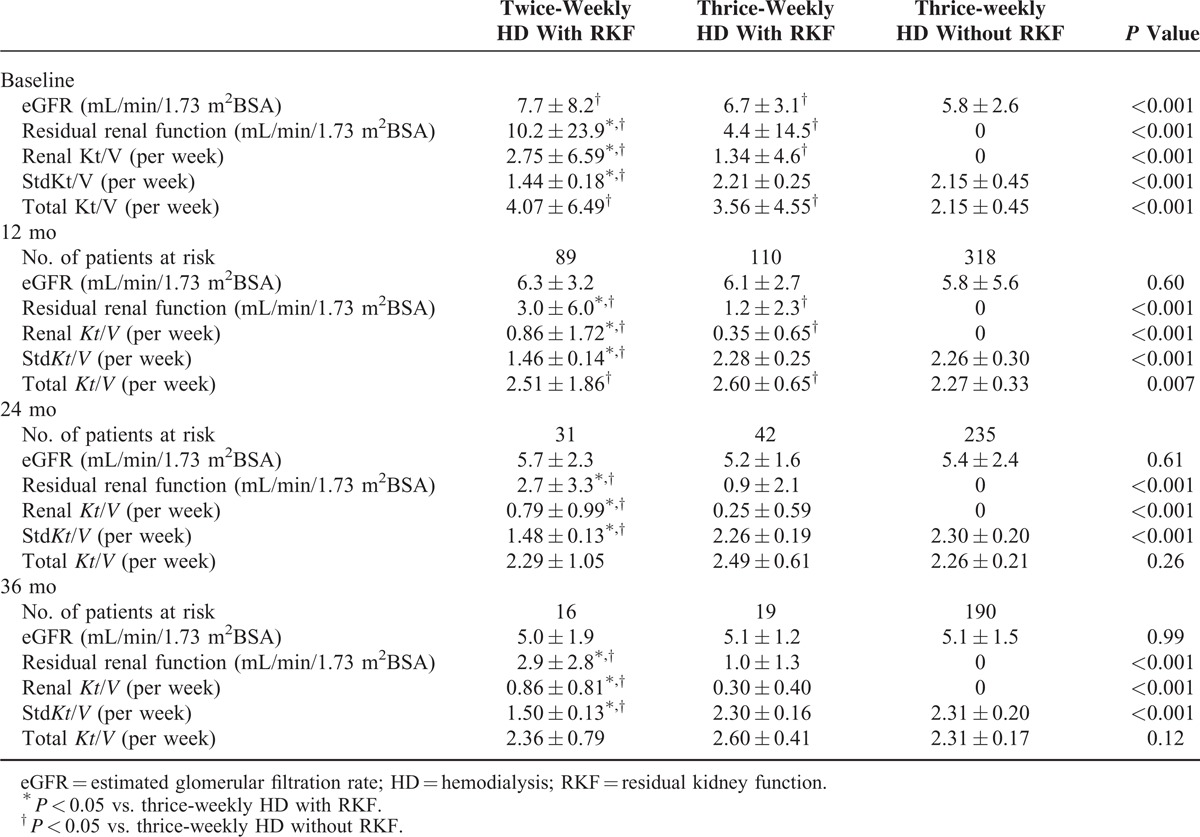
Comparison of Residual Renal Function and Dialysis Adequacy Between Patients With and Without RKF

### Fluid Balance and nPCR Values in Patients Receiving Twice-Weekly and Thrice-Weekly HD

Dry weight did not differ between the 3 patient groups at any follow-up point (Table 3). Ultrafiltration was significantly lower during the entire follow-up in patients with RKF undergoing twice-weekly HD. However, this group showed a greater 24-h urine volume than either of the 2 thrice-weekly HD groups. The total fluid removal (ultrafiltration plus 24-h urine volume) in patients with RKF undergoing twice-weekly HD did not differ from that in patients with RKF undergoing thrice-weekly HD. The net fluid balance (postdialysis weight minus dry weight) did not differ between the 3 groups at baseline, but became significantly lower in patients undergoing twice-weekly HD than in patients with or without RKF receiving thrice-weekly HD. Patients with RKF receiving twice-weekly HD showed a higher baseline nPCR than patients without RKF undergoing thrice-weekly HD, but after 24 months of follow-up a lower nPCR was observed in patients with RKF undergoing twice-weekly HD than in patients with RKF undergoing thrice-weekly HD. Compared with patients without RKF receiving thrice-weekly HD, total fluid removal was greater in patients with RKF undergoing thrice-weekly HD up to 24 months of follow-up.

**TABLE 3:**
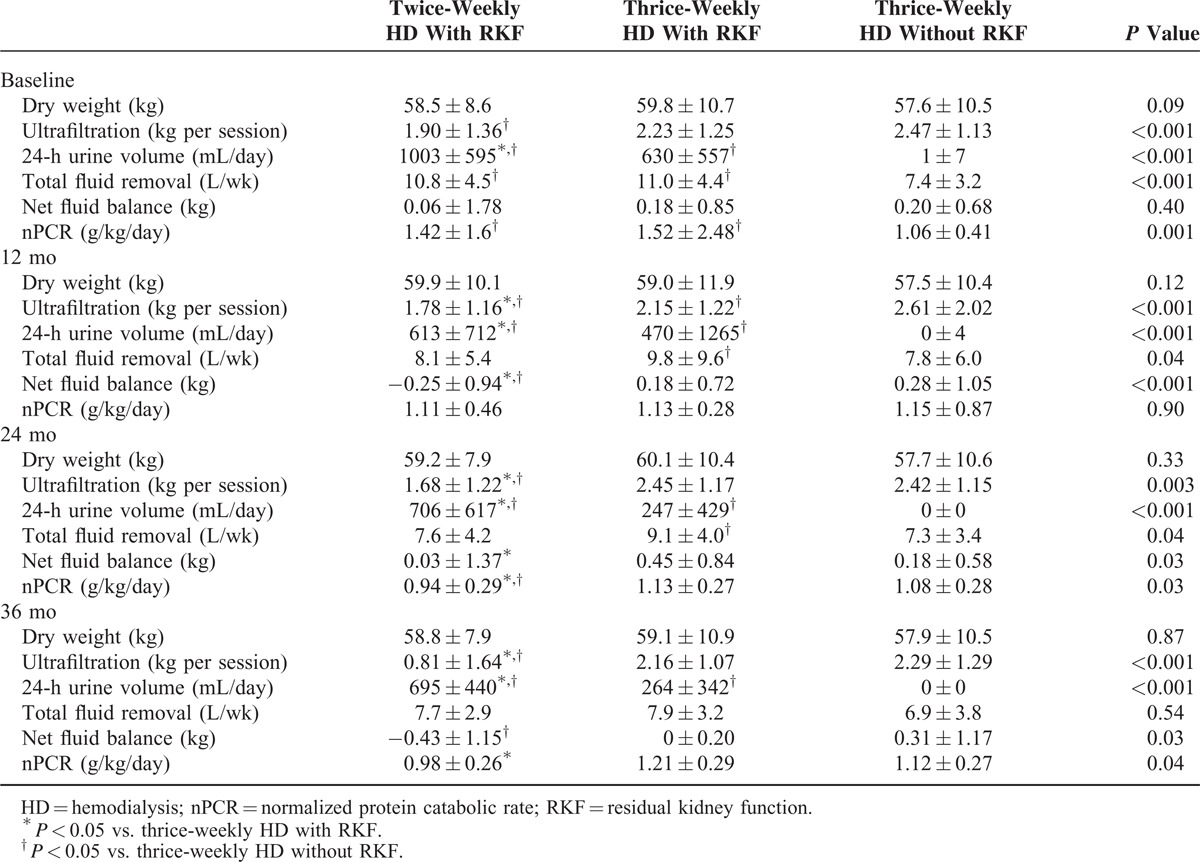
Comparison of Dialysis Characteristics Between Patients With and Without RKF

### Follow-Up Laboratory Data in Twice-Weekly and Thrice-Weekly HD Patients

We compared the follow-up laboratory data between 3 patients groups. The hemoglobin level, serum level of albumin and potassium was not different during entire follow-up (Table 4). The serum bicarbonate level at 24-month follow-up and calcium phosphate product at 36-month follow-up was significantly lower in patients with RKF receiving twice-weekly HD than patients without RKF receiving thrice-weekly HD.

**TABLE 4:**
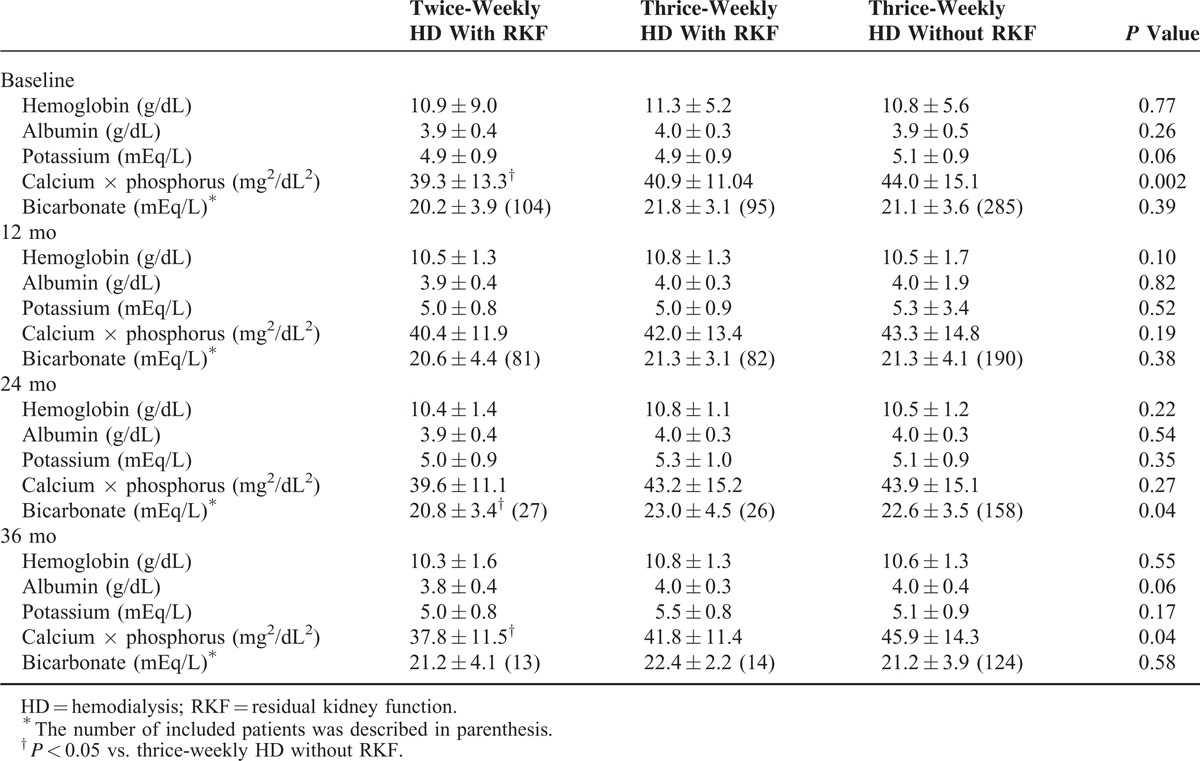
Comparison of Follow-Up Laboratory Data Between Patients With and Without RKF

### Patient Death and CVE Hospitalization in Patients Receiving Twice-Weekly and Thrice-Weekly HD

**FIGURE 1:**
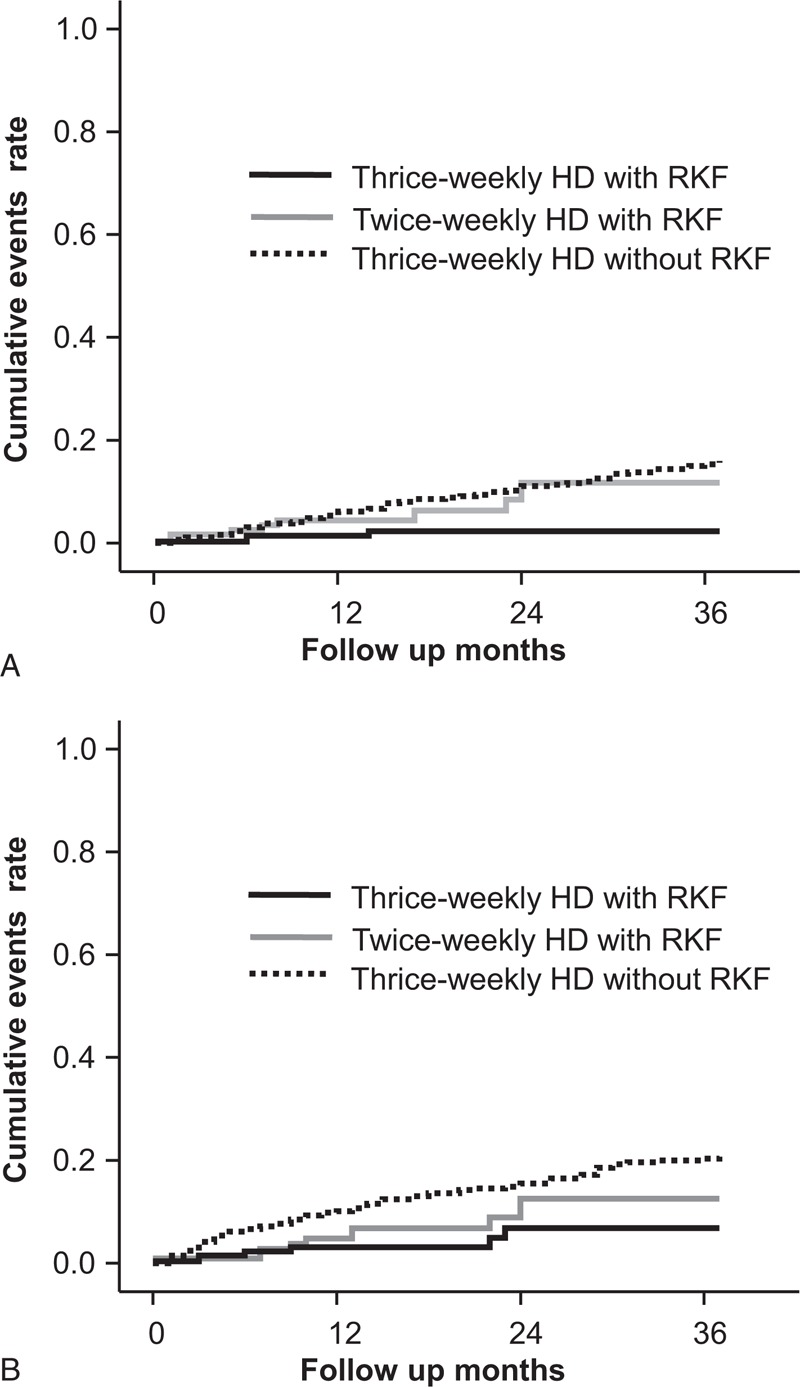
Cumulative incidence curves for patient survival rate (A) and cardiovascular hospitalization events (B). The cumulative death rate was significantly lower in patients with RKF undergoing thrice-weekly HD than in patients without RKF undergoing thrice-weekly HD (*P* < 0.001) or in patients with RKF undergoing twice-weekly HD (*P* = 0.02). The lowest cumulative event rate for CVE hospitalization was observed in patients with RKF undergoing thrice-weekly HD (*P* = 0.01). CVE = cardiovascular event; HD = hemodialysis; RKF = residual kidney function.

We compared the cumulative event rate for patient death and CVE hospitalization. The number of patient deaths was 3 (2.2%) in patients with RKF undergoing thrice-weekly HD, 8 (7.1%) in patients with RKF undergoing twice-weekly HD, and 59 (13.6%) in patients without RKF undergoing thrice-weekly HD (*P* < 0.001, Table 5). The cumulative event rate for patient death at the 3-year follow-up was 11.8% in patients with RKF receiving twice-weekly HD, which was not higher than that in patients without RKF receiving thrice-weekly HD (16.0%; *P* = 0.57) (Figure 1A). Of the 3 groups, patients with RKF undergoing thrice-weekly HD had the lowest cumulative event rate for patient death (6.8%, *P* = 0.002). In patients with RKF, those undergoing twice-weekly HD had a significantly higher event rate than those receiving thrice-weekly HD (*P* = 0.02).

**TABLE 5:**
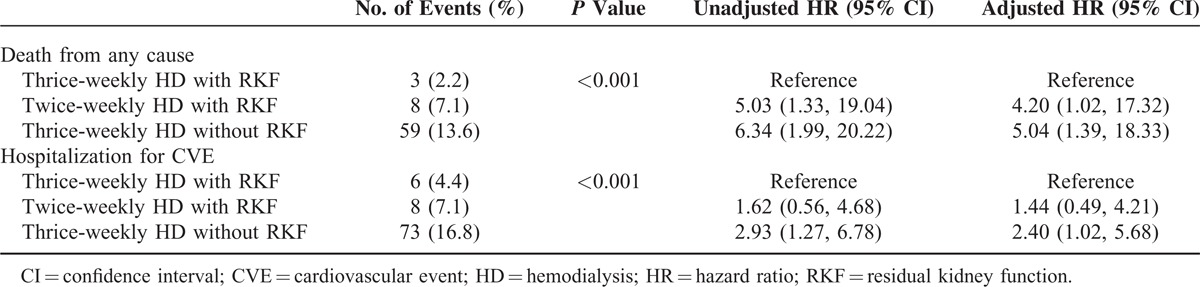
Incidence and Hazard Ratios of Deaths and CVE Based on Status of HD Frequency and RKF

The patients with RKF undergoing thrice-weekly HD had 6 (4.4%) CVE, and 8 (7.1%) and 73 (16.8%) of CVE was observed in patients with RKF receiving twice-weekly HD and in patients without RKF receiving thrice-weekly HD, respectively (*P* < 0.001). The lowest cumulative event rate for CVE hospitalization was observed in patients with RKF receiving thrice-weekly HD (*P* = 0.01) (Figure 1B). For patients with RKF, the CVE event rate was not significantly higher in those receiving twice-weekly HD than in those receiving thrice-weekly HD (*P* = 0.35). Compared with patients without RKF undergoing thrice-weekly HD, patients with RKF undergoing twice-weekly HD had a noninferior event rate (*P* = 0.11).

### Impact of HD Frequency on Mortality and Morbidity in Patients Receiving Twice-Weekly and Thrice-Weekly HD

The hazard ratio (HR) for mortality is described in Table 5. In a multivariate Cox proportional hazards model, compared with patients with RKF undergoing thrice-weekly HD, patients with RKF undergoing twice-weekly HD had a higher mortality risk (HR, 4.20; 95% CI, 1.02–17.32, *P* = 0.04), and greater HR was also observed in patients without RKF undergoing thrice-weekly HD (HR, 5.04; 95% CI, 1.39–18.33, *P* = 0.014). When the patients without RKF undergoing thrice-weekly HD were considered a reference group, patients with RKF undergoing twice-weekly HD had a noninferior risk for mortality (HR, 0.83; 95% CI, 0.34–2.01, *P* = 0.68).

The HR for CVE hospitalization was evaluated in comparison with patients with RKF undergoing thrice-weekly HD. Patients with RKF undergoing twice-weekly HD did not show an independently increased risk (HR, 1.44; 95% CI, 0.49–4.21, *P* = 0.50). The patients without RKF undergoing thrice-weekly HD had a 2.40-fold risk for CVE hospitalization (95% CI, 1.02–5.68, *P* = 0.04). Compared with patients without RKF undergoing thrice-weekly HD, patients with RKF undergoing twice-weekly HD had a noninferior risk (HR, 0.60; 95% CI, 0.28–1.29, *P* = 0.19).

## DISCUSSION

A reduction in the frequency of HD may be considered in patients with RKF. In our study, patients with RKF undergoing twice-weekly HD had a higher urine volume, greater RKF and greater renal *Kt*/*V* than patients with RKF undergoing thrice-weekly HD, and their total *Kt*/*V* similar to that of patients undergoing thrice-weekly HD. In addition, net fluid balance was significantly lower in patients with RKF undergoing twice-weekly HD. Despite these positive parameters, our study revealed that patients with RKF undergoing twice-weekly HD had an increased risk for mortality compared with patients with RKF undergoing thrice-weekly HD. This finding suggests that decisions about HD frequency should consider not only RKF, but also other risk factors that may influence mortality in patients undergoing HD.

RKF is a critical determinant for urea removal and it might allow the reduction of the mortality in patients undergoing twice-weekly HD.^13,16^ However, the results of our study were discordant with results from previous studies, which showed the noninferiority of a twice-weekly regimen.^6,7^ It is likely that this discrepancy derived from the regular 24-h urine follow-up. In the present study, the RKF was monitored regularly by urine collection, and the data for patients were censored when RKF disappeared. In addition, we also censored the events for primary and secondary outcomes if the dialysis frequency was changed by a clinician. Therefore, our study is more useful for evaluating the actual clinical outcomes when a twice-weekly HD regimen and substantial RKF is maintained in HD patients.

It has been reported that twice-weekly HD dialysis therapy was associated with better preservation of RKF than thrice-weekly HD.^17,18^ Our study also demonstrated that the RKF was significantly higher at baseline in patients undergoing twice-weekly HD, and that it remained higher at all follow-up points. This advantage in RKF for patients in this study receiving twice-weekly HD contributed to urea clearance. Total *Kt*/*V* was similar in patients with RKF undergoing twice-weekly and thrice-weekly HD, while std*Kt*/*V* was lower in patients with RKF receiving twice-weekly HD. These findings suggest that twice-weekly HD treatment can achieve a sufficient dialysis dose, similar to that of thrice-weekly HD treatment, if RKF is appropriately preserved.

HD patients receiving infrequent HD treatment are at risk of high interdialytic weight gain and hypervolemic status.^3^ However, in the present study, the dry weight of the 3 patient groups did not differ, and the net fluid balance was lower in patients with RKF receiving twice-weekly HD. It was thought that a higher urine volume successfully prevented the volume overload that can occur with infrequent HD treatment. However, the greatest total fluid removal was observed in patients with RKF undergoing thrice-weekly HD, and nPCR was significantly higher in these patients. We presumed that a greater total fluid removal in patients with RKF undergoing thrice-weekly HD was associated with greater intake of caloric and protein foods, which led to better nutritional status.^19^

Infrequent HD treatment could expose patients to several risk factors for CVE including hypervolemia, hyperkalemia, and higher levels of calcium phosphate product.^2,3,20^ However, RKF significantly contributes to reducing the risk of CVE in HD patients.^16,21,22^ In the present study, patients undergoing twice-weekly HD had a noninferior survival rate for CVE compared with patients without RKF undergoing thrice-weekly HD. In addition, patients with RKF undergoing twice-weekly HD did not demonstrate the electrolyte derangements or fluid excess that can occur with infrequent HD treatment. These findings suggest that RKF in patients undergoing twice-weekly HD successfully offsets the risk of CVE.

While our study demonstrated the noninferior mortality of patients undergoing twice-weekly HD compared with patients without RKF undergoing thrice-weekly HD, patients with RKF undergoing twice-weekly HD had a significantly higher mortality than patients with RKF undergoing thrice-weekly HD. We observed a lower nPCR at the 24-month and 36-month follow-ups in patients with RKF undergoing twice-weekly HD. We presumed that this could be one of the reasons why patients with RKF undergoing twice-weekly HD had worse outcomes, because protein intake is an important risk factor for mortality in HD patients.^23,24^ Therefore, we suggest that HD patients with unacceptable nPCR values should not be considered candidates for a twice-weekly HD regimen, while the requirements for dialysis dose, fluid balance, and electrolyte control are fully satisfied.

Our study has several limitations. First, this study was not a randomized trial. Therefore, we could not reach a firm conclusion that patients with RKF undergoing thrice-weekly HD had better outcomes than patients with RKF undergoing twice-weekly HD. Second, a monitoring of RKF is performed once a year, not at 3-month intervals. Third, patients with RKF receiving twice-weekly HD had baseline biases such as shorter duration of dialysis, greater standard deviation of RKF, higher rates of low flux dialyzer use, and higher incidence of catheter use. Fourth, one might discuss 24 h of 300 mL or less might also be an appropriate cut-off for inclusion in the group without RKF. Fifth, additional measure of equivalent renal urea clearance (EKR) would be helpful to include the urea clearance of RKF to total clearance, because it appreciates dialysis dose more than std*Kt*/*V*.^25,26^

In conclusion, patients undergoing twice-weekly HD had noninferior outcomes for mortality and CVE hospitalization compared with patients without RKF undergoing thrice-weekly HD. However, patients with RKF undergoing twice-weekly HD had an increased risk of mortality compared with patients with RKF undergoing thrice-weekly HD, although RKF successfully supported adequate urea removal and fluid and electrolyte balance. We suggest that decisions about infrequent HD should be made based not only on RKF, but also on other risk factors such as nPCR.
